# The shock index for pre-hospital identification of trauma patients with early acute coagulopathy and massive bleeding

**DOI:** 10.1186/s13054-015-0839-3

**Published:** 2015-03-27

**Authors:** Pierre Pasquier, Clément Dubost, Brice Malgras, Kevin Kearns, Stéphane Mérat

**Affiliations:** Intensive Care Unit, Bégin Military Teaching Hospital, 69 avenue de Paris, 96160 Saint-Mandé, France; Department of Surgery, Val-de-Grâce Military Teaching Hospital, 74 boulevard de Port Royal, 75005 Paris, France

We read with interest the article by Tonglet and colleagues [1], who evaluated the efficacy of the Trauma-Induced Coagulopathy Clinical Score (TICCS) to discern between major trauma patients who require damage control resuscitation and those who do not. TICCS, an easily and rapidly computed score by paramedics at a trauma scene, is based on three clinical components: general severity of the trauma, blood pressure (BP), and extent of tissue injuries. We would like to go further into the discussion and propose that shock index (SI) could be a more reliable component than BP for TICCS calculation. SI is defined as the ratio of heart rate (HR) to systolic BP. This easily calculable score has been demonstrated to be a pragmatic and useful guide for diagnosing acute hypovolemia, even in the presence of normal HR and BP. SI has been shown to correlate with other indices of end-organ perfusion, such as central venous oxygen saturation and arterial lactate concentration [2]. In place of HR or systolic BP alone, SI has been used as a marker for severity of injury and poor outcome in trauma patients. Rady and colleagues [3] found that, in a cohort of 275 adult patients, SI of more than 0.9 was associated with worse outcomes in trauma patients. Finally, a pre-hospital SI for trauma correlates with measures of hospital resource use and mortality [4,5]. We would like to know whether the authors could give their opinion regarding the calculation of SI for pre-hospital identification of trauma patients with early acute coagulopathy and massive bleeding, including its potential usefulness for TICCS evaluation.

## Abbreviations

BP: Blood pressure
HR: Heart rate
SI: Shock index
TICCS: Trauma-Induced Coagulopathy Clinical Score
